# Ethoxyquin Inhibits the Progression of Murine Ehrlich Ascites Carcinoma through the Inhibition of Autophagy and LDH

**DOI:** 10.3390/biomedicines9111526

**Published:** 2021-10-23

**Authors:** Fekria Tayel, Magdy E. Mahfouz, Afrah F. Salama, Mohammed A. Mansour

**Affiliations:** 1 Biochemistry Division, Department of Chemistry, Faculty of Science, Tanta University, Tanta 31527, Egypt; fekria.tayel@yahoo.com (F.T.); afrahsalama@yahoo.com (A.F.S.); 2 Zoology Department, Faculty of Science, Kafrelsheikh University, Kafr El Sheikh 33516, Egypt; mmahfouz4@yahoo.co.uk; 3 Cancer Biology and Therapy Lab, Division of Human Sciences, School of Applied Sciences, London South Bank University, London SE1 0AA, UK

**Keywords:** ethoxyquin, LDH, glycolysis, autophagy, cisplatin, Ehrlich ascites carcinoma

## Abstract

Cancer cells exhibit an increased glycolysis rate for ATP generation (the Warburg effect) to sustain an increased proliferation rate. In tumor cells, the oxidation of pyruvate in the Krebs cycle is substituted by lactate production, catalyzed by LDH. In this study, we use ethoxyquin (EQ) as a novel inhibitor to target LDH in murine Ehrlich ascites carcinoma (EAC) and as a combination therapy to improve the therapeutic efficacy of the conventional chemotherapy drug, cisplatin (CIS). We investigated the anti-tumor effect of EQ on EAC-bearing mice and checked whether EQ can sustain the anti-tumor potential of CIS and whether it influences LDH activity. Treatment with EQ had evident anti-tumor effects on EAC as revealed by the remarkable decrease in the expression of the anti-apoptotic gene *Bcl-2* and by a significant increase in the expression of apoptotic genes (*BAX* and *caspase-3*). EQ also caused a significant decrease in the autophagic activity of EAC cells, as shown by a reduction in the fluorescence intensity of the autophagosome marker. Additionally, EQ restored the altered hematological and biochemical parameters and improved the disrupted hepatic tissues of EAC-bearing mice. Co-administration of EQ and CIS showed the highest anti-tumor effect against EAC. Collectively, our findings propose EQ as a novel inhibitor of LDH in cancer cells and as a combinatory drug to increase the efficacy of cisplatin. Further studies are required to validate this therapeutic strategy in different cancer models and preclinical trials.

## 1. Introduction

Glycolysis is the process in which the entire glucose molecule is broken into two pyruvate molecules. In aerobic conditions, glycolysis is followed by the citric acid cycle, which allows cells to “burn” pyruvate molecules and produce chemical energy in the form of ATP [1]. In cancer cells, glucose molecules are only partially broken down in glycolysis and then undergo the conversion of pyruvate molecules into lactate in a biochemical reaction catalyzed by lactate dehydrogenase (LDH) [2]. An abnormal reliance on glycolysis as the only source of ATP production, even in the presence of oxygen, is evident in many cancer cells and is commonly called the ‘Warburg effect’.

Some of the most important HIF-1α target genes involved in tumorigenesis are associated with glucose metabolism. High glycolytic flux is the outcome of HIF-1α upregulation. Under hypoxic conditions, lactate dehydrogenase catalyzes the formation of lactate as the end product of anaerobic glycolysis [3]. Activation of an HIF-linked molecular cascade causes tumor aggressiveness by triggering anaerobic metabolism (LDH-5 overexpression) or by activating genes related to angiogenesis (VEGF) [4].

Recent research indicates that utilizing aerobic glycolysis and changes in the metabolic environment may prevent immune cells from recognizing cancer cells and eradicating them [5]. These metabolic changes in cancer cells may also activate oncogenes that permit cancer cells to avoid programmed cell death (apoptosis). Moreover, the products of aerobic glycolysis may be utilized to form products that aid cancer cells to survive and grow [6].

Lactate dehydrogenase, which catalyzes the production of lactate in the last step of the glycolytic pathway, is a central player in the Warburg effect [2]. The structure of LDH is well-preserved in different species, with only slight changes in its amino acid sequence across species [7]. This structural similarity of LDH among species provides the rationale for designing small-molecule inhibitors to modulate its catalytic function in cells. LDH is a cytoplasmic enzyme with five isozymes (LDH1-5) which are differentially expressed in different tissues. LDH’s active site contains catalytically active residues, e.g., His-193, Asp-168, Arg-171, Thr-246, and Arg-106 [8]. The function of LDH, specifically LDH-5, is modified in cancer cells to sustain glycolysis and ATP production through lactate, even in the presence of oxygen. In most invasive tumors, LDH-5 (also called LDH-A) is upregulated; therefore, the inhibition of LDH-5 inhibits tumor progression and invasiveness [9,10]. For instance, the inhibition of LDH (by classical LDH inhibitors, such as oxamate) promotes the infiltration and activity of immune cells in non-small-cell lung cancer (NSCLC) [11]. Treatment by oxamate increases the infiltration of activated CD8+ T cells in the tumor and enhances the therapeutic effects of immune checkpoint inhibitors such as pembrolizumab and turns tumors into ‘hot tumors’. LDH levels can also serve as a prognostic and diagnostic marker of tumor progression in different types of malignancies, including cutaneous lymphoma. LDH-5 is also useful to predict the response of cancer patients to radiotherapy and chemotherapy, and to evaluate metastatic cancer patients [12,13,14].

In cancer cells, an increased proliferation rate needs a high metabolic turnover because of the induced stress on cells that continue to grow and divide [1]. Therefore, cancer cell homeostasis needs the activation of autophagy to fight metabolic stressors, which permits the recycling of energy sources such as ATP, which are necessary to sustain survival [15]. Moreover, autophagy activation plays a role in chemo and/or radiation resistance. Autophagy inhibition in tumor cells has been found to enhance the potential of anticancer agents [16,17]. In the present study, we performed a comprehensive molecular docking study to select novel LDH inhibitors that can be utilized in combination with the chemotherapeutic drug cisplatin (CIS) to improve its therapeutic efficiency and decrease associated adverse effects. We investigated the novel anti-tumor potential of ethoxyquin (EQ) via the inhibition of LDH activity in a murine EAC model, which is referred to as an undifferentiated carcinoma, and is originally hyper diploid, has high transplantable capability, no regression, rapid proliferation, and a shorter life span. We also elucidated the possible underlying mechanisms associated with the EQ-induced inhibition of cancer cells with a special focus on apoptosis and autophagy.

## 2. Materials and Methods

### 2.1. Computational Molecular Docking

Docking server (https://www.dockingserver.com; accessed on 1 August 2020) was utilized to perform the in silico molecular docking analysis of the LDH protein (Protein Data Bank (PDB): 4OJN) and different selected ligands, as shown in Table 1. The partial charges of Gasteiger were added to the ligand atoms, non-polar hydrogen atoms were merged, and rotatable bonds were defined. The calculations were carried on out on ethoxyquin (EQ) interacting with LDH (https://www.rcsb.org/structure/4OJN; accessed on 15 August 2020). AutoDock tools were used for the addition of essential hydrogen atoms and solvation parameters. Van der Waals and electrostatic energies were calculated and docking simulations were performed by a Lamarckian genetic algorithm (LGA) and Solis–Wets methods. After 250,000 maximum energy evaluations, each docking test was elucidated from 10 different runs set to terminate.

### 2.2. Chemicals

Ethoxyquin, 0.4%, trypan blue solution, an autophagy assay kit for fluorometric tests and ferric tripyridyltriazine (FeIII-TPTZ) chemicals were purchased from Sigma Aldrich (St. Louis, MO, USA). LDH assay kits were purchased from Mybiosource USA (Inc. P.O. San Diego, CA, USA). Cisplatin was obtained from a ready-to-use infusion. An RNeasy Mini Kit (# 74104), a Quantiscript Reverse Transcription Kit (#205310), and QuantiTect SYBR Green PCR Kits were purchased from Qiagen (Hilden, Germany). Kits for biochemical assays were purchased from the Bio Diagnostic medical company (Giza, Egypt).

### 2.3. Transplantation of Tumor Cells

EAC cells have been collected from a donor mouse purchased from the National Cancer Institute, Cairo University, Egypt, by injection of 1 mL of sterile PBS into the peritoneal cavity of the donor mouse to dilute the liquid tumor. Diluted EAC cells were withdrawn from this mouse and were suspended in sterile isotonic saline, and the viable EAC cells were counted using the trypan blue exclusion method and adjusted at 0.5 × 10^6^ EAC cells/mouse [18].

### 2.4. Mice Experimental Design

Normal, female, Swiss albino mice, aged 6–8 weeks, weighing 18–20 g, were brought from the National Cancer Institute (Cairo University, Cairo, Egypt), kept in standard laboratory experimental conditions (temperature 25 ± 2 °C, relative humidity 55% ± 5%, balanced diet, and free access to water), and left for 2 weeks before experimentation, to adapt to laboratory conditions. All mice experiments were performed according to the Scientific Procedures Act, 1986, and the ARRIVE guidelines, and approved by the animal ethical committees (Faculty of Science, Tanta University, Tanta, Egypt).

**Figure 1:**
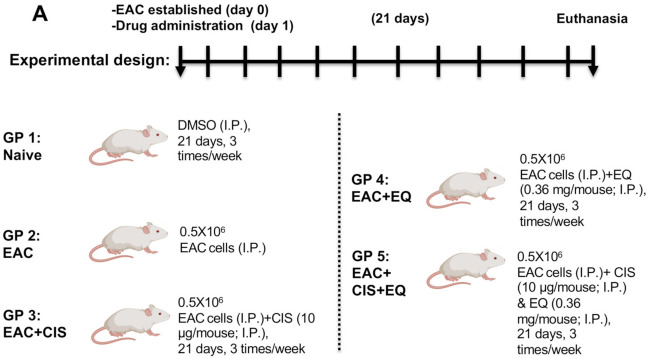
(**A**) Ehrlich ascites carcinoma experimental design. EAC: Ehrlich ascites carcinoma; EQ: ethoxyquin; CIS: cisplatin; and DMSO: dimethyl sulfoxide.

One hundred mice were randomly assigned to five groups (*n* = 20/group) (Figure 1). GP 1: Naïve group was injected by IP (intraperitoneal injection) with DMSO (vehicle) (3.6 µL/mouse) for 21 days—3 times/week. GP 2: EAC-bearing control group was inoculated with EAC cells (0.5 × 10^6^/mouse) by IP at day 0. GP 3: CIS-treated EAC group was treated with CIS (10 µg/mouse) by IP for 21 days—3 times/week. GP 4: EQ-treated EAC group was given EQ (0.36 mg/mouse) by IP for 21 days—3 times/week. GP 5: CIS + EQ-treated EAC group was treated with EQ (0.36 mg/mouse) and CIS (10 µg/mouse) by IP for 21 days—3 times/week. The treatments started the day after the inoculation of EAC cells.

### 2.5. Sampling

The mice were monitored for any changes in eating, excretion, or behavior. At the end of the experimental period, ten mice from each group were euthanized, and ascitic fluid was collected from the peritoneal cavity by syringe. The tumor volume was measured by taking it into a graduated centrifuge tube, and the collected peritoneal ascitic fluid was centrifuged at 1300× *g* for 10 min. Supernatants were separated, and cells were washed three times with PBS and then resuspended in sterile isotonic saline (PBS) to count them. The tumor cell count was performed in a Neubauer hemocytometer, and the total viable and non-viable EAC cells were counted using the trypan blue exclusion method (Bothara et al., 2015). The cells were stored at −20 °C for further downstream processing.

Blood samples were collected at the end of the experiment (21 days) in either vacutainer tubes containing heparin (for hematological assays) or plain centrifuge tubes, left to clot, and centrifuged at 3000 rpm for 10 min for serum separation, which was stored at −20 °C till they were used for biochemical assays. Following euthanization by decapitation under ether anesthesia, liver samples were immediately removed from mice (*n* = 10/group), weighed, and divided into two parts. One part was kept in a 10% neutral buffer, formalin, for histopathological examination, and the second was frozen at −20 °C until it was used for biochemical assays. The remaining mice in each group (*n* = 10/group) were kept alive to calculate the increase in life span (ILS%) and mean survival time (MST).

### 2.6. Cell Count and Viability

To evaluate the cell count and viability, EAC cells were stained using 0.4% trypan blue staining and counted in a hemocytometer. The percentage of viable cells was quantified using the formula: ((total number of cells − number of trypan-blue-positive cells)/total number of cells) × 100.

### 2.7. Lactate Dehydrogenase Activity

Lactate dehydrogenase enzyme activity was measured by using a lactate dehydrogenase activity colorimetric assay kit (Biovision, Milpitas, CA, USA). LDH reduces NAD to NADH, which then interacts with a probe to produce a color (λmax = 450 nm). This color’s intensity is measured before and after the incubation time to estimate the amount of NADH produced per minute. The amount of LDH which catalyzes the conversion of lactate into pyruvate to generate 1.0 μmole of NADH per minute at 37 °C is the unit calculated (U). The kit quantifies LDH activity in a variety of biological samples, such as serum, plasma, cells, and tissue extracts. In this study, we measured LDH activity in EAC cells, serum, and liver tissue homogenate.

### 2.8. RNA Extraction from EAC Cells and Liver Tissue

We extracted RNA from purified EAC cells and liver tissue cells. We used liver tissue cells to determine antioxidant gene expression and EAC cells isolated from the ascetic fluid withdrawn from the peritoneal cavity to determine apoptotic gene expression. A Total RNA Purification Kit was used for the extraction of pure RNA following the manufacturer’s protocol (Qiagen/Germany, cat# 74104 and 74106). Briefly, a lysis buffer that contains guanidine isothiocyanate (high concentration) was used to homogenize biological samples (EAC cells and liver tissues). This helps RNA to bind to the silica membrane and β-mercapto-ethanol to effectively inactivate RNases in the lysate. Ethanol is added to the lysate, mixed well, and then loaded onto a purification column [19]. While the lysate was spun through the column, the guanidine isothiocyanate and ethanol cause RNA to bind to the silica membrane. After that, wash buffers were used to wash the column to effectively remove impurities from the membrane. Finally, an elution process was performed to purify RNA with nuclease-free water under low-ionic-strength conditions.

### 2.9. qPCR Analysis

Total RNA was extracted from EAC cells and liver tissues by using an RNeasy Mini Kit containing DNase 1, as described previously [20,21]. Quantiscript reverse transcriptase was used for the synthesis of cDNA from 4 µg of total RNA in a final volume of 40 μL. The synthesized cDNA was utilized as a template to estimate the relative expression of apoptotic genes, *caspase-3*, *BAX*, and *Bcl-2*, as well as the antioxidant genes, *SOD*, *GSH*, and *CytP450*, utilizing a StepOnePlus Real-Time PCR System (qPCR in triplicate) (Applied Biosystem, Waltham, MA, USA) and gene-specific primers (oligo dT primers) designed by Primer Premier 5.0 software. The following primers sequences were purchased: F: 5′-CATGCCAAGAGGGAAACACCAGAA-3′ and R: 5′-GTGCTTTGCATTCTTGGATGAGGG-3′ for Bcl-2; F: 5′-TTCATTATTCAGGCCTGCCGAGG-3′ and R: 5′-TTCTGACAGGCCATGTCATCCTCA-3′ for caspase-3; F: 5′-GGCTGGACACTGGACTTCCT-3′ and R: 5′-GGTGAGGACTCCAGCCACAA-3′ for BAX; F: 5′-GGGAAGCTGTTGTCCCAAG-3′ and R: 5′-CAAGGGGAGGTAAAAGAGAGC-3′ for SOD1; F: 5′-GGTTCGAGCCCAATTTTACA-3′ and R: 5′-CCCACCAGGAACTTCTCAAA-3′ for GSH-Px1; F: 5′-CTGCAGACACTACTACTACA-3′ and R: 5′-ATCCGAGTCACTGCTCTCAG-3′ for CytP450. β-actin with primer sequence; F: 5′ ACTATTGGCAACGAGCGGTT 3′ and R: 5′ CAGGATTCCATACCCAAGAAGGA 3′ was utilized as an internal control to calculate the fold change in target genes. A total of 25 µL of PCR mix included 12.5 µL of 2× quantitect SYBR Green qPCR Master Mix, 2 µL of cDNA template (20 ng/μL), 1 µL of forward primer (10 μM), 1 µL of reverse primer (10 μM), and 8.5 µL of nuclease-free water. The thermal cycle was 95 °C for 10 min, followed by 40–45 cycles at 95 °C for 15 s and 60 °C for 1 min, as recommended by the manufacturer’s protocol. The critical thresholds (Ct) quantities of target genes were normalized with the quantities (Ct) of β-actin using the 2^−∆∆Ct^ method, as described previously [22,23]. Calculation of 2^−∆∆Ct^ determined the fold change, and the control group was used as the reference control group as for *GSH*, *SOD*, and *Cyp19A1* gene expression; however, the fold change in the treatment groups was measured relative to the EAC group as in *Caspase 3*, *Bcl2*, and *BAX* genes. The experiments were performed in triplicates among study groups.

### 2.10. Detection of Autophagy by Flow Cytometry

A flow cytometer was used for the detection of EAC fluorescent autophagosomes. A proprietary fluorescent autophagosome marker (λex = 333/λem = 518 nm) was utilized within an autophagy assay kit (Sigma Aldrich, USA). One hundred microliters of each ascetic fluid of EAC-bearing control mice and treated groups was added to 1 mL of phosphate-buffered saline then centrifuged for 5 min at 21 °C. An autophagosome detection reagent (4,6-diamidino-2-phenylindole (DAPI stain)) was diluted with Dulbecco’s Modified Eagle Medium at 1/10 dilution, and then 4 µL of this reagent was added to cells in each well and the mixture was incubated at 37 °C with 5% CO2 for 30 min. The excess dye was removed by washing with a wash buffer and then centrifuged for 5 min at 21 °C for removal of the excess dye. The washing step was repeated twice.

One hundred microliters of phosphate-buffered saline was added to the last pellet and the fluorescence was determined (Ex. 360 nm, Em. 520 nm), where ex: fluorescent excitation (blue), em: fluorescent emission (green) in blue configuration, with an Attune Flow Cytometer (Applied Biosystem, USA).

### 2.11. Hematological and Biochemical Assays

Hemoglobin (Hb) content, white blood cell count (WBC), red blood cell count (RBC), and platelets were estimated using standard automated procedures. Measurement of the liver enzyme activities (aspartate aminotransferase (AST), alanine aminotransferase (ALT), gamma-glutamyl transferase (GGT), and albumin) was performed in serum, utilizing local diagnostic kits. Total protein in serum was measured by utilizing the Biuret reagent. Measurement of the hepatic concentration of total antioxidant capacity (TAC), a lipid peroxidation biomarker (malondialdehyde: MDA), glutathione-S-transferase (GST), hepatic total protein contents, and the activity of the antioxidant enzyme catalase (CAT) was performed in liver tissues following the manufacturer protocols and as described previously [24,25].

### 2.12. Histopathological Investigation

Liver specimens were excised from mice after dissection and fixed in 4% paraformaldehyde solution (PFA) at room temperature for 2 h. Hepatic tissues were then washed using phosphate-buffered saline (PBS) twice, dehydrated with serial dilutions of ethanol, cleared in xylene, and embedded in paraffin (56 °C for 24 h). Tissues embedded in paraffin wax blocks were cut using microtome, collected on gelatin chrom/alum-coated glass slides, deparaffinized, and stained with hematoxylin and eosin prior to investigation under light microscopy [26].

### 2.13. Statistical Analysis

The mean ± standard error (SE) was used for the representation of the statistical analysis data. Significant differences among treated groups and controls were estimated by two-way analysis of variance (ANOVA) employing GraphPad Prism Software 7. All groups were compared with each other to show the significant impact of treatment groups. The significance for the statistical analysis was set at *p* ≤ 0.05.

## 3. Results

### 3.1. Intensive Molecular Docking Analysis

**Figure 2:**
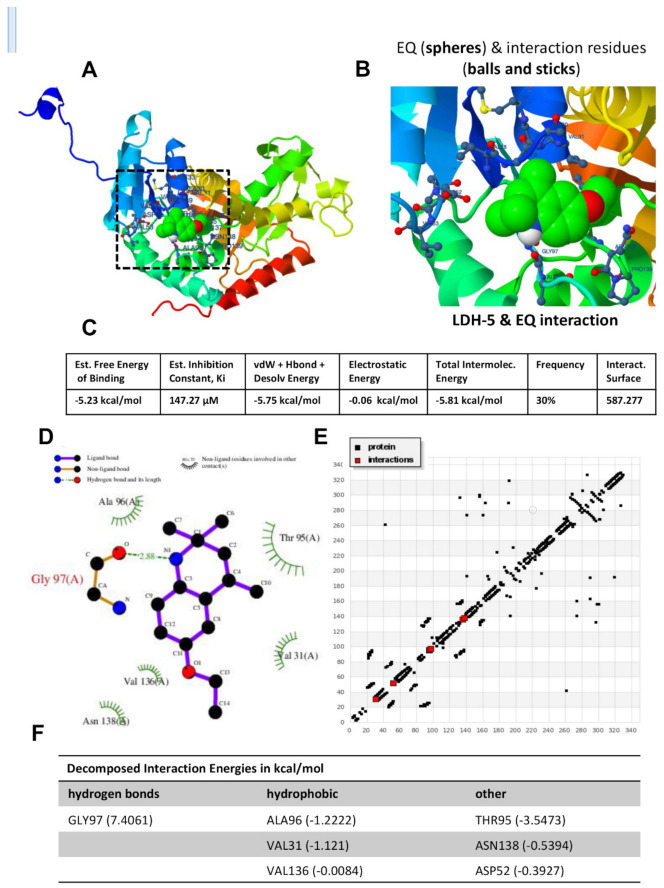
Molecular docking analysis of LDH and ethoxyquin. (**A**) The 3D modeling of the crystal structure of LDH (PDB: 4OJN) with ethoxyquin interacting with active residues of LDH. (**B**) Magnified image of the interaction of ethoxyquin with active residues at LDH’s active site (e.g., Gly–97, Val–31, and Ala–98). (**C**) The estimated free energy of binding, inhibition constant (ki), vdW + H-bond + Desolv energy, electrostatic energy, and total intermolecular energies of ethoxyquin and LDH molecular docking. (**D**) The 2D plot of ethoxyquin interacting with LDH active residues (e.g., Thr–95, Gly–97, and Val–136) through ligand, non-ligand, and hydrogen bonding (HB). (**E**) HB plot with active residues involved in LDH–ethoxyquin interactions. (**F**) The decomposed interaction energies with hydrogen and hydrophobic bandings between EQ and different active residues. EQ, ethoxyquin; LDH, lactate dehydrogenase.

To find potent and novel inhibitors of human LDHA, found in muscles, we performed an in silico molecular docking analysis for LDHA (PDB: 4OJN) with several quinoline compounds, as shown in Table 1, using the molecular docking server (https://www.dockingserver.com; accessed on 1 August 2020). The screening analysis revealed ethoxyquin as a potent inhibitor of LDH, as it has the highest free energy of binding and the lowest inhibition constant (Figure 2A,B). Ethoxyquin interacts with active residues (e.g., Gly-97, Val-31, and Ala-98) in the active site of LDH (Figure 2B). The binding energies are estimated as follows: free energy of binding (−5.23 kcal/mol), vdW + Hbond + Desolv energy (−5.75 kcal/mol), electrostatic energy (−0.06 kcal/mol), and total intermolecular energy (−5.81 kcal/mol). The inhibition constant (Ki) of ethoxyquin with LDH is estimated to be 147.27 µM (Figure 2C). The hydrogen blotting of the interaction revealed that ethoxyquin interacts with Ala-96, Gly-97, Val-136, Asn-138, Val-31, Thr-95, and Asp-52 (Figure 2D,E). Figure 1F shows Gly-97 to hydrogen bond with ethoxyquin, while Ala-96, Val-31, and Val-136 to have hydrophobic interactions with ethoxyquin. Other amino acids (Thr-95, Asn-138, and Asp-52) have other types of bonding with ethoxyquin (Figure 2F).

### 3.2. The Effect of EAC and Treatments on Body Weight and Hematological Parmeters

The liver weight of untreated EAC mice (2.38 ± 0.12 g, *n* = 10) significantly (*p* ≤ 0.001) increased, and was 1.45-fold more than that of normal control mice (1.64 ± 0.09 g, *n* = 10). Treatment of EAC mice (*n* = 10/group) with CIS and EQ alone or in combination resulted in a significant 0.74 (*p* ≤ 0.001)-, 0.75 (*p* ≤ 0.01)-, or 0.56 (*p* ≤ 0.0001)-fold decrease in liver weight, respectively, as compared to untreated EAC mice. The relative liver weight of G2 showed a significant (*p* ≤ 0.0001) decrease as compared to the Gp 1 (naïve) group. Treatment with CIS and EQ alone or in combination demonstrated a significant (*p* ≤ 0.01) (*p* ≤ 0.0001) (*p* ≤ 0.05) increase in relative liver weights as compared to EAC-bearing group (Table 2).

The data presented in Table 2 reveal that the hematological parameters (Hb% and RBC and WBC count) of untreated EAC mice significantly (*p* ≤ 0.0001) decreased and platelet count significantly increased (*p* ≤ 0.0001) compared to normal control mice.

Administration of CIS and EQ alone or in combination resulted in a significant increase in hemoglobin (*p* ≤ 0.01) (*p* ≤ 0.0001) (*p* ≤ 0.0001), RBCs (*p* ≤ 0.05) (*p* ≤ 0.01) (*p* ≤ 0.0001), and WBCs (*p* ≤ 0.0001) (*p* ≤ 0.0001) (*p* ≤ 0.01), and a significant decrease in platelet (*p* ≤ 0.0001) (*p* ≤ 0.0001) (*p* ≤ 0.0001) count compared to the EAC group.

### 3.3. The Anti-Tumor Effect of EQ against EAC

To evaluate the anti-tumor effects of EQ against EAC, changes in body weight, liver weight, relative liver weight (Table 2), tumor length, width and volume, fluid volume, count of tumor cells, MST, and ILS% of treated groups were observed (Table 3). The body weight of untreated EAC mice (31.0 ± 0.71 g, *n* = 10) significantly (*p* ≤ 0.0001) increased by 1.57-fold more than that of normal control mice (19.8 ± 0.58 g, *n* = 10). To confirm our hypothesis, we treated EAC-bearing mice with CIS and EQ alone and in combination to examine whether the combined treatment gives better anti-tumor potential. Treatment of EAC mice (*n* = 10/group) with CIS and EQ alone or in combination resulted in a significant 0.74 (*p* ≤ 0.0001)-, 0.75 (*p* ≤ 0.0001)-, or 0.56 (*p* ≤ 0.0001)-fold decrease in body weight, respectively, as compared to untreated EAC mice.

Administration of CIS and EQ alone or in combination led to a significant decrease in viable (%) of tumor cell count as well as tumor length, width, volume, and fluid volume in comparison with EAC mice. Treatment of EAC mice with EQ showed no significant change in non-viability and the total number of tumor cells in comparison to the EAC-bearing group. The CIS-treated group (*p* < 0.05) and EQ + CIS-treated group (*p* < 0.01) induced a significant increase in non-viable tumor cell count in comparison to EAC group. The treatment of EAC mice with CIS group only (*p* < 0.001) and EQ + CIS group (*p* < 0.0001) induced a significant decrease in total tumor cell count in comparison to the EAC group (Table 3).

On the other hand, treatment of mice (*n* = 10/group) with CIS and EQ alone or in combination significantly increased ILS with a rate of 120%, 153.3%, and 190%, respectively, as compared to the EAC group. Additionally, naive mice showed a significant increase in ILS with a rate of 296.67% as compared to the EAC group. Administration of CIS and EQ alone or in combination led to a notable increase in MST with 33, 38, and 43.4 days, respectively, as compared to the EAC group (15 days). Additionally, normal mice showed a significant increase in MST with 59.5 days as compared to EAC group. From the three treatments, the combination group shows the most improvement in MST and ILS as compared to the EAC group (Table 3).

### 3.4. Effect of CIS and/or EQ on Biochemical Parameters of EAC-Bearing Mice

The administration of CIS and EQ alone or in combination resulted in a significant (*p* ≤ 0.0001) decrease in AST, ALT, and GGT as compared to the EAC group (Table 2). The total protein in serum and albumin of the EAC group significantly (*p* ≤ 0.0001 and *p* ≤ 0.001) decreased as compared to normal control mice. Administration of EQ alone or in combination resulted in a significant (*p* ≤ 0.05) increase in albumin as compared to the EAC group, while administration of CIS alone results in no significant difference as compared to the EAC group. Administration of CIS and EQ alone or in combination resulted in a significant (*p* ≤ 0.0001) increase in serum total protein as compared to the EAC group (Table 2).

The EAC group showed a significant (*p* ≤ 0.0001) decrease in hepatic total protein, catalase, GST, and TAC, and a significant (*p* ≤ 0.0001) increase in hepatic MDA as compared to the normal group. Treatment with CIS and EQ alone or in combination showed a significant increase in hepatic total protein (*p* ≤ 0.01) (*p* ≤ 0.0001) (*p* ≤ 0.0001), catalase (*p* ≤ 0.0001), TAC (*p* ≤ 0.0001), and GST (*p* ≤ 0.01) (*p* ≤ 0.0001) (*p* ≤ 0.0001), respectively, and showed a significant decrease in hepatic MDA (*p* ≤ 0.0001) as compared to the EAC group (Table 4).

Treatment with CIS and EQ alone or in combination showed a significant increase in total protein (*p* ≤ 0.05) (*p* ≤ 0.0001) (*p* ≤ 0.0001), GST (*p* ≤ 0.0001), TAC (*p* ≤ 0.0001), and catalase (*p* ≤ 0.01) (*p* ≤0.0001) (*p* ≤ 0.0001), respectively, in EAC cells, and showed a significant decrease in MDA (*p* ≤ 0.0001) as compared to the untreated group (Table 4).

### 3.5. Effect of CIS and/or EQ on LDH Activity in Serum, Liver Tissue, and EAC Cells

**Figure 3:**
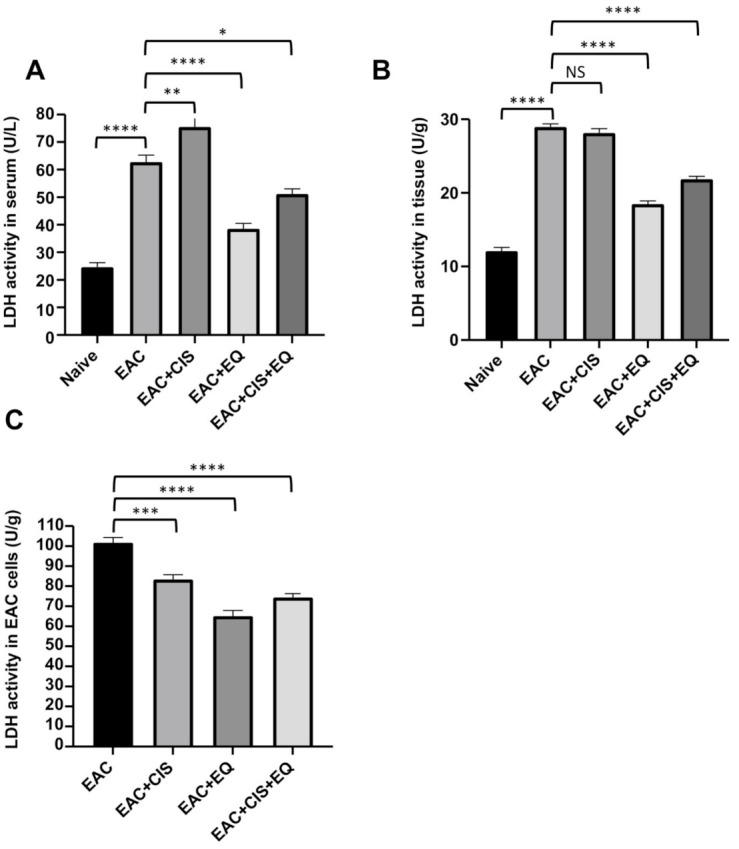
Inhibition of LDH activity by ethoxyquin. An LDH activity assay was performed by an LDH colorimetric assay in each experimental group (enzymatic unit (U): amount of LDH which catalyzes the conversion of lactate into pyruvate to generate 1.0 μmole of NADH per minute at 37 °C). (**A**) LDH activity in serum. (**B**) LDH activity in liver tissue. (**C**) LDH activity in EAC cells. *, *p* < 0.05; **, *p* < 0.01; ***, *p* < 0.001; and ****, *p* < 0.0001 in comparison with respective groups. NS, nonsignificant; EAC, Ehrlich ascites carcinoma; LDH, lactate dehydrogenase; EQ: ethoxyquin; and CIS, cisplatin.

The EAC-bearing group showed a significant (*p* ≤ 0.0001) increase in LDH activity in serum and liver tissue, as compared to the normal group (Figure 3A,B). Administration of EQ alone or in combination resulted in a significant decrease in LDH activity, in serum (*p* ≤ 0.0001) (*p* ≤ 0.05), as well as in liver tissue (*p* ≤ 0.0001) (*p* ≤ 0.0001), respectively, with the lowest reduction in the EQ-treated group, as compared to the untreated EAC-bearing group (Figure 3A,B). Treatment with CIS resulted in a significant (*p* ≤ 0.01) increase in serum (Figure 3A) and a non-significant change in LDH activity in liver tissue as compared to the untreated EAC-bearing group (Figure 3B). Treatment with CIS and EQ alone or in combination resulted in a significant (*p* ≤ 0.001) (*p* ≤ 0.0001) (*p* ≤ 0.0001) decrease in LDH activity in EAC cells, respectively, with the lowest reduction in the EQ-treated group, as compared to the untreated EAC-bearing group (Figure 3C).

The CIS-treated group (*p* ≤ 0.0001) (*p* ≤ 0.0001) and the EQ + CIS-treated group (*p* ≤ 0.01) (*p* ≤ 0.0001) showed a significant increase in LDHA activity in serum and liver tissue, respectively, as compared to the EQ-treated group (Figure 3A,B). The CIS-treated group (*p* ≤ 0.001) showed a significant increase in LDHA activity in EAC cells as compared to the EQ-treated group (Figure 3C). The EQ + CIS-treated group showed no significant change in LDHA activity in EAC cells compared to the EQ-treated group (Figure 3C). The CIS-treated group showed a significant (*p* ≤ 0.0001) increase in LDHA activity in serum and liver tissue, and showed no significant change in LDHA activity in EAC cells as compared to the EQ + CIS-treated group (Figure 3).

### 3.6. Effect of CIS and/or EQ on the Relative Expression of Antioxidant Genes (SOD, GSH, and CytP450) in Liver Tissue

**Figure 4:**
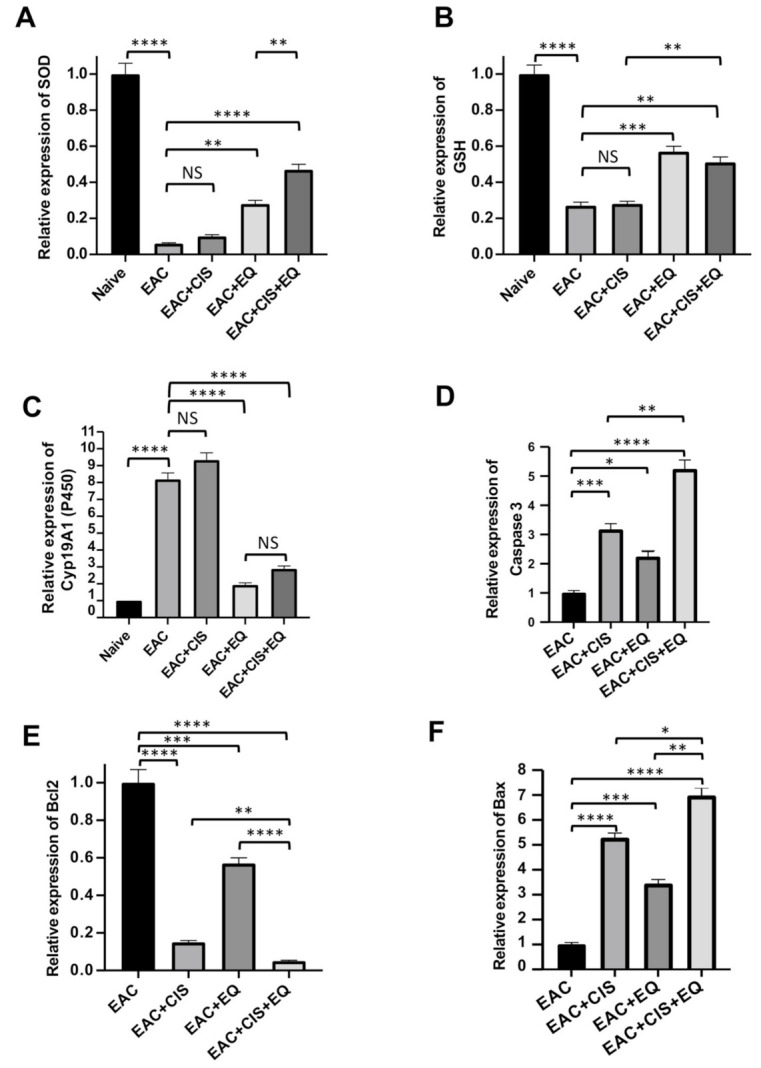
Relative expression of antioxidant genes and apoptotic genes. (**A**–**C**) Relative expression of *SOD*, *GSH*, and *Cyp19A1(P450)*, respectively. (**D**–**F**) Relative expression of caspase-3, Bcl-2, and BAX, respectively. The experiments were performed in triplicates. *, *p* < 0.05; **, *p* < 0.01; ***, *p* < 0.001; and ****, *p* < 0.0001 in comparison with respective groups. NS, nonsignificant; EAC, Ehrlich ascites carcinoma; EQ, ethoxyquin; and CIS, cisplatin.

The EAC-untreated group showed a significant (*p* ≤ 0.0001) decrease in antioxidant gene expression (*SOD* and *GSH*) and a significant (*p* ≤ 0.0001) increase in CytP450 as compared to the normal group (Figure 4). Results revealed that treatment with EQ alone or in combination resulted in a significant (*p* ≤ 0.01) (*p* ≤ 0.0001) increase in *SOD* gene expression, respectively (Figure 4A), as well as a significant (*p* ≤ 0.001), (*p* ≤ 0.01) increase in GSH gene expression, respectively, as compared with the untreated EAC group (Figure 4B). The same treatment resulted in a significant (*p* ≤ 0.0001 and *p* ≤ 0.0001) decrease in CytP450 gene expression, respectively, as compared with the untreated EAC group (Figure 4C). However, treatment with CIS results in a non-significant change in *SOD*, *GSH*, and *CytP450* gene expression (Figure 4A–C).

The CIS-treated group showed a significant decrease in *SOD* (*p* ≤ 0.05) and *GSH* (*p* ≤ 0.001) gene expression, respectively, and showed a significant (*p* ≤ 0.0001) increase in *CytP450* gene expression as compared to the EQ-treated group. The EQ + CIS-treated group showed a significant (*p* ≤ 0.05) increase in *SOD* gene expression and showed no significant change in *GSH* and *CytP450* gene expression as compared to the EQ-treated group. The CIS-treated group showed a significant decrease in SOD (*p* ≤ 0.0001) and *GSH* (*p* ≤ 0.01) gene expression and showed a significant (*p* ≤ 0.0001) increase in *CytP450* gene expression as compared to the EQ + CIS-treated group (Figure 4A–C).

### 3.7. Effect of CIS and/or EQ on the Relative Expression of Apoptotic Genes (Bcl-2, Caspase-3, and BAX Genes) in EAC Cells

Real-time PCR (RT-PCR) was used to detect the relative expression of apoptosis regulatory genes, *Bcl-2*, *caspase-3*, and *BAX*, which reflect the changes in transcription levels of these genes in EAC cells after treatment with CIS and/or EQ in comparison to EAC mice. Our results revealed a significant (*p* ≤ 0.001) (*p* ≤ 0.05) (*p* ≤ 0.0001) upregulation of caspase-3 gene expression following treatment with CIS and EQ alone and in combination, respectively with the highest expression in the combined group as compared to untreated EAC group. EQ + CIS-treated group showed a significant (*p* ≤ 0.0001) upregulation of *caspase-3* gene expression as compared to the EQ-treated group. The CIS-treated group showed no significant change in the *caspase-3* gene expression level as compared to the EQ-treated group (Figure 4D).

Additionally, the administration of CIS and EQ alone or in combination resulted in a significant (*p* ≤ 0.0001) (*p* ≤ 0.001) (*p* ≤ 0.0001) downregulation of the *Bcl-2* gene, respectively, with the lowest expression in the combined group, as compared to the untreated EAC group. The CIS-treated group (*p* ≤ 0.001) and the EQ + CIS-treated group (*p* ≤ 0.0001) showed a significant downregulation of *Bcl-2* gene expression as compared to the EQ-treated group (Figure 4E).

The same treatments resulted in a significant (*p* ≤ 0.0001) (*p* ≤ 0.001) (*p* ≤ 0.0001) upregulation of the *BAX* gene with the highest expression in the combined group, as compared to the untreated EAC group. The CIS-treated group (*p* ≤ 0.01) and the EQ + CIS-treated group (*p* ≤ 0.0001) showed a significant upregulation of *BAX* gene expression as compared to the EQ-treated group (Figure 4F).

The CIS-treated group showed a significant upregulation of caspase-3 (*p* ≤ 0.001) and *BAX* (*p* ≤ 0.01) gene expression and showed no significant change in *Bcl-2* gene expression as compared to the EQ + CIS-treated group (Figure 4D–F).

### 3.8. Effect of CIS and/or EQ on the Autophagic Activity of EAC Cells

**Figure 5:**
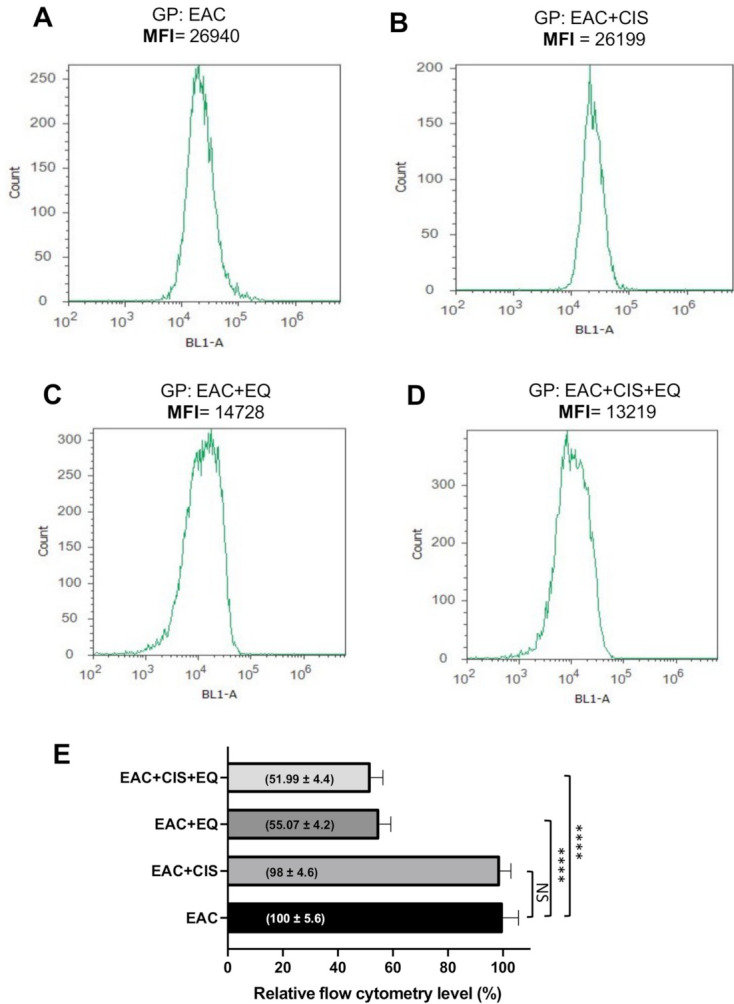
Flow cytometry analysis of autophagic activity of EAC cells (**A**–**D**) by measuring the fluorescence intensity of the autophagosome marker in different experimental groups. (**E**) Relative flowcytometry level (%). ****, *p* < 0.0001 in comparison with respective groups. NS, nonsignificant; EAC, Ehrlich ascites carcinoma; EQ, ethoxyquin; CIS, cisplatin; and MFI, mean fluorescence intensity.

Flowcytometry analysis of the autophagic marker (fluorescence intensity of autophagosome marker) (Figure 4A–D) revealed a significant decrease (*p* ≤ 0.0001) in autophagic activities in both EQ- and EQ + CIS-treated groups as compared to the untreated EAC group. However, there was no significant change following treatment with CIS alone. The CIS-treated group showed a significant increase (*p* ≤ 0.0001) in autophagic activities as compared to the EQ-treated group. The EQ + CIS-treated group showed no significant change in autophagic activities as compared to the EQ-treated group. The CIS-treated group showed a significant increase in autophagic activities (*p* ≤ 0.0001) as compared to the EQ + CIS-treated group (Figure 5E).

### 3.9. Histopathological Findings

**Figure 6:**
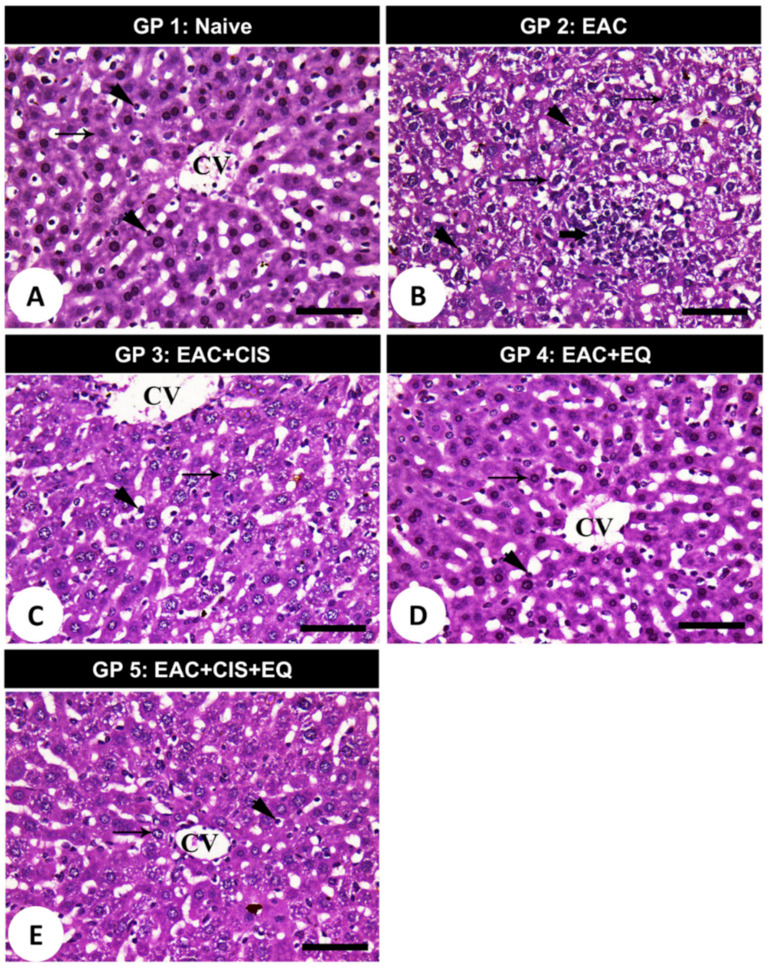
Photomicrographs of liver sections stained with hematoxylin and eosin (H&E). (**A**) Negative control group displayed normal hepatic architecture with polyhedral hepatocytes (arrow) with centrally located nuclei. The hepatocytes were arranged in strands alternating with blood sinusoids forming a network around a central vein. Kupffer cells (arrowhead) were distributed within the blood sinusoids. (**B**) Hepatic tissue of mice in the EAC group (GP 2) shows a distinct disruption of hepatocyte arrangement. Hepatocytes have a clear to foamy cytoplasm with intranuclear cytoplasmic inclusions (thin arrows) and sinusoidal infiltration of clumps of EAC tumor cells mixed with lymphocytes (thick arrows). Additionally, Kupffer cells increased significantly within the sinusoids (arrowheads). (**C**) Liver sections with good improvement in the hepatic architecture except in slight hepatocyte vacuolations (arrow) and moderate distribution of Kupffer cells (arrowhead) in the CIS-treated group. (**D**) Liver sections revealed better improvement in the hepatic architecture with healthy hepatocytes (arrow) and normal distribution of Kupffer’s cells (arrowhead) in the EQ-treated group. (**E**) Liver sections with good improvement in the hepatic architecture except in slight hepatocyte vacuolations (arrow) and normal distribution of Kupffer cells (arrowhead) in the combination treatment group. Scale bars = 12.5 mm (**A**), 25 mm (**B**,**D**,**E**), and 50 mm (**C**).

Histopathological examination of mice liver tissues of different studied groups demonstrated the following: the negative control group displayed a normal hepatic architecture with polyhedral hepatocytes (Figure 6A; arrow) with centrally located nuclei. The hepatocytes were arranged in strands alternating with blood sinusoid forming a network around a central vein. Kupffer cells (Figure 6A; arrowhead) were distributed within the blood sinusoids. Liver sections of the EAC group displayed distinct disruption of the characteristic cord-like arrangement of the hepatocytes. Hepatocytes had a clear to foamy cytoplasm with intranuclear cytoplasmic inclusions (Figure 6B; thin arrows), sinusoidal infiltration of clumps of Ehrlich tumor cells mixed with lymphocytes (Figure 6B; thick arrows). Kupffer cells increased significantly within the sinusoids (Figure 6B; arrowheads). Liver sections of EAC treated with CIS showed good improvement in the hepatic architecture except in slight hepatocyte vacuolations (Figure 6C; arrow) and moderate distribution of Kupffer cells (Figure 6C; arrowhead). Liver sections of EAC treated with EQ revealed better improvement in the hepatic architecture with healthy hepatocytes (Figure 6D; arrow) and normal distribution of Kupffer’s cells (Figure 6D; arrowhead). Liver sections of EAC treated with the combination of CIS and EQ showed good improvement in the hepatic architecture except in slight hepatocyte vacuolations (Figure 6E; arrow) and normal distribution of Kupffer cells (Figure 6E; arrowhead).

## 4. Discussion

Discovery of effective cancer therapeutics to successfully target hard-to-cure cancers through their metabolic regulators is still an active area of research. In the present study, we revealed that treatment with ethoxyquin (a novel LDH inhibitor) significantly increased the anti-cancer effect of cisplatin in Ehrlich ascites carcinoma (EAC). EAC models resemble human tumors and are sensitive to chemotherapy since they are undifferentiated cancer cells with a rapid growth rate. This effect is attributed to the role of ethoxyquin in the induction of apoptosis and the inhibition of autophagy. Intraperitoneal administration of CIS and/or EQ significantly reduced the ascites (tumor volume) and tumor cell count, and increased the percentage of trypan-blue-positive stained dead cells as compared to tumor-bearing mice, an effect which is attributed to the induction of apoptosis by CIS and/or EQ. To assess whether the administration of EQ alone or combined with CIS can overcome the adverse effects of CIS when given alone, we evaluated the changes in some hematological parameters (Hb content and RBC, WBC, and PLT count). EQ alone and/or combination treatment mostly alleviated the remarkable alterations in the hematological as well as biochemical parameters.

In contrast to normal cells, cancer cells are characterized by the development of a hypoxic and aggressive phenotype associated with invasiveness and resistance to conventional cancer treatments, such as chemotherapy and radiotherapy [27]. In contrast to normal cells, cancer cells have a metabolic shift from oxidative phosphorylation (OXPHOS) to cytoplasmic glycolysis. This metabolic reprogramming in cancer cells provides rapid ATP production to maintain energy status, increase synthesis of precursors for macromolecule biosynthesis, and maintain an appropriate cellular redox system [16]. LDH catalyzes the conversion of the end product of glycolysis, pyruvate, to lactate, and thereby modulates the metabolic reprogramming of tumor cells and contributes to the metabolic alterations required for tumor progression [28].

Overexpression of LDH-5 has been frequently reported in various glycolytic cancers, such as colorectal, prostate, pancreatic, lung, gastric, and endometrial cancers, as well as melanoma and non-Hodgkin lymphoma [29,30,31,32,33,34,35,36,37]. LDH-5 overexpression confers the growth advantages of highly glycolytic cancer cells and correlates with an aggressive phenotype and a poor prognosis [37]. Of note, hypoxia-inducible factor (HIF)-1, c-Myc, HER2/neu, and forkhead box protein (FOXM) 1 bind to the promoter to increase its expression, glucose utilization, and lactate production [38,39,40]. The inhibition of LDH is currently emerging as an attractive and potentially safe treatment option to disrupt the energy supply in cancer. Several studies have been performed to investigate the effect of LDH inhibition on the invasiveness, metastasis, and proliferation potential of cancer cells [41,42]. In a renal cancer xenograft mouse model, LDH-5 silencing by short hairpin RNA (shRNA) markedly decreased tumor formation [43]. Further, the use of small-molecule inhibitors of LDH-5 increased the production of mitochondrial reactive oxygen species (ROS) and oxidative stress, resulting in cancer cell death [44]. In addition, combination of the LDH inhibitor (oxamate) with taxol or trastuzumab showed a synergistic effect and sensitized taxol- or trastuzumab-resistant breast cancer cells by promoting cellular apoptosis [45].

The first developed LDH inhibitors were originally designed to inhibit the LDH isoform in malarial *Plasmodium falciparum* and showed low species specificity, and thereby could inhibit human LDH as well [46]. Notably, there are three groups of current LDH inhibitors based on their binding sites: inhibitors binding (i) to the pyruvate- and/or nicotinamide-binding pocket; (ii) to the adenosine- and pyruvate/nicotinamide-binding pockets; and (iii) with unconfirmed binding modes [47]. For example, oxamate is a well-known substrate-competitive inhibitor of human LDH (Ki values of 94.4 and 136 mM against human LDH-5 and LDH-1, respectively) [48]. Other successful chemical inhibitors of LDH are highly polar, elongated, and ‘obese’ molecules. Bioisosteric replacement of some residues in these compounds is suggested to improve cellular permeability and/or bioavailability of these compounds [47]. An analogue, the N-hydroyxindole class of LDH inhibitors, is also investigated as an anticancer agent [49]. Bifunctional LDH inhibitors which can bind to both substrate-binding and cofactor regions of the active site are also being discovered and are showing great potential [50].

In the current study, EQ enhances the anti-tumor potential of CIS against EAC-bearing mice through the activation of apoptosis (programmed cell death) and the inhibition of autophagic flux of cancer cells. The evasion of apoptosis mechanisms plays a key role in tumor pathogenesis, protecting cancer cells from oxidative stress and hypoxia as the tumor mass spreads. Therefore, effective induction of apoptosis using novel therapeutics may be a key strategy for blocking recurrence and cancer metastasis [51]. Autophagy is a lysosomal degradation pathway that is essential for survival, differentiation, development, and homeostasis in living cells [52]. Autophagy is upregulated in response to different types of stress that disturb cellular homeostasis, involving low cellular ATP levels, nutrient and growth factor deprivation, hypoxic conditions, endoplasmic reticulum (ER) stress, pathogen entry, or anticancer drug treatment [52]. Similarly, when tumor cells are exposed to hypoxic stress, they induce autophagy to protect cells from death [53]. Therefore, successful anticancer agents are designed to activate apoptotic and inactivate autophagy pathways in highly glycolytic cancers.

## 5. Conclusions

In conclusion, from an experimental standpoint, we provide ethoxyquin as a novel LDH inhibitor and an autophagic inhibitor for cancer cells, which has the potential to be used in combination with traditional chemotherapy, e.g., CIS. Administration of EQ significantly enhanced the anti-tumor effects of CIS on cancer cells through the inhibition of tumor-promoting autophagic flux and LDH activity. These findings potentiate the importance of ethoxyquin as a novel LDH inhibitor and anticancer agent that can be used to treat cancer in an EAC model. Further investigations are required to investigate the impact of this inhibitor and the combination therapy on different types of cancer cells.

## Figures and Tables

**Table 1:** Molecular docking of FDA-approved quinoline derivatives with LDH (PDB: 4OJN).

Compound	Est. Free Energy of Binding (Kcal/mol)	Est. Inhibition Constant, Ki (µM)	VdW + Hbond + Desolv Energy (Kcal/mol)	Electrostatic Energy (Kcal/mol)	Total Intermolec. Energy (Kcal/mol)	Frequency (%)	Interact. Surface
Ethoxyquin (1,2-dihydro-2,2,4-trimethyl-6-Ethoxyquinoline)	−5.23	147.27	−5.75	−0.06	−5.81	30%	587.277
Ethyl 4-hydroxy-7-trifllouromethyl-3-quinolinecarboxylate	−5.13	174.28	−5.97	−0.24	−6.21	20%	670.941
2,2,4-Trimethyl-6-Trityl-1,2-Dihydro-quinoline	−5.09	185.74	−5.63	−0.02	−5.65	40%	597.442
5-chloro-7-iodo-8-quinolinol	−4.97	226.28	−5.30	0.03	−5.27	30%	439.625
6-Methoxy-2,2,4-Trimethyl-8-Trityl-1,2-Dihydro-Quinoline	−4.42	577.84	−5.96	−0.90	−6.85	10%	779.012

**Table 2:** Biochemical and hematological parameters in blood in different groups of experimental mice.

Parameters	Gp 1 (Naive)	Gp 2 (EAC)	Gp 3 (EAC + CIS)	Gp 4 (EAC + EQ)	Gp 5 (EAC + CIS + EQ)
Body weight (g)	19.8 ± 0.58	31.0 ± 0.71 ***	23.0 ± 0.71 ^####^	23.2 ± 0.86 ^####^	20.4 ± 0.81 ^####^
Liver weight (g)	1.64 ± 0.09	2.38 ± 0.12 ***	1.7 ± 0.07 ^##^	1.74 ± 0.12 ^##^	1.46 ± 0.07 ^####^
Relative liver weight (g/100 g body weight)	8.28 ± 0.34	5.74 ± 0.13 ****	7.39 ± 0.26 ^##^	8.02 ± 0.36 ^####^	7.16 ± 0.22 ^###^
Hb% (g/dL)	12.02 ± 0.19	7.14 ± 0.22 ****	8.49 ± 0.32 ^##^	10.16 ± 0.41 ^####^	10.62 ± 0.3 ^####^
WBCs (10^3^/µL)	7.69 ± 0.58	16.66 ± 0.70 ****	10.62 ± 0.64 ^####^	11.02 ± 0.50 ^####^	13.12 ± 0.44 ^##^
RBCs (10^6^/µL)	7.76 ± 0.1	5.0 ± 0.23 ****	5.88 ± 0.16 ^##^	6.34 ± 0.29 ^#^	6.65 ± 0.17 ^####^
Platelets (10^6^/µL)	0.43 ± 0.02	0.86 ± 0.03 ****	0.5 ± 0.03 ^####^	0.48 ± 0.03 ^####^	0.52 ± 0.01 ^####^
Plasma AST (U/L)	37.32 ± 1.07	85.31 ± 1.24 ****	70.78 ± 1.27 ^####^	50.88 ± 1.20 ^####^	45.23 ± 1.66 ^####^
Plasma ALT (U/L)	26.96 ± 0.95	60.90 ± 1.21 ****	44.86 ± 0.91 ^####^	41.32 ± 0.56 ^####^	37.27 ± 0.63 ^####^
Plasma total protein (g/dL)	5.30 ± 0.17	3.32 ± 0.17 ****	6.48 ± 0.13 ^####^	5.35 ± 0.2 ^####^	5.44 ± 0.21 ^####^
Plasma albumin (U/L)	3.49 ± 0.19	2.48 ± 0.14 ***	3.02 ± 0.08 ^#^	3.03 ± 0.07 ^#^	3.13 ± 0.1 ^#^
Plasma GGT (U/L)	18.93 ± 0.33	46.52 ± 0.88 ****	36.29 ± 0.54 ^####^	25.12 ± 0.70 ^####^	33.36 ± 0.37 ^####^

Values are represented as mean ± SE, *n* = 10 for each group. Means with different superscript asterisks in the same row differ significantly at (*p* < 0.05). ***, *p* < 0.001, and ****, *p* < 0.0001 in comparison of the EAC group (Gp 2) with the normal control group (Gp 1), and ^#^, *p* < 0.05; ^##^, *p* < 0.01; ^###^, *p* < 0.001; and ^####^, *p* < 0.0001 in comparison of treatment groups with the EAC group (Gp 2). EAC, Ehrlich ascites carcinoma; CIS, cisplatin; EQ, ethoxyquin; ALT, alanine transaminase; AST, aspartate transaminase; GGT, gamma glutamyl transferase; ALP, alkaline phosphatase; Hb%, hemoglobin concentration; RBCs, red blood cells; and WBCs, white blood cells.

**Table 3:** Physiological parameters that showing the anti-tumor effect of EQ in different groups of experimental mice.

Parameters	Gp 1 (Naive)	Gp 2 (EAC)	Gp 3 (EAC + CIS)	Gp 4 (EAC + EQ)	Gp 5 (EAC + CIS + EQ)
Tumour width (cm)	-	5.6 ± 0.29	4.0 ± 0.18 ^###^	3.8 ± 0.3 ^###^	3.6 ± 0.19 ^####^
Tumour length (cm)	-	5.29 ± 0.19	3.95 ± 0.20 ^###^	3.9 ± 0.19 ^###^	3.82 ± 0.13 ^####^
Fluid volume (mL)	-	1.59 ± 0.12	0.56 ± 0.05 ^####^	0.83 ± 0.07 ^####^	0.66 ± 0.04 ^####^
Tumour volume (cm^3^)	-	83.58 ± 8.99	31.35 ± 1.59 ^####^	28.99 ± 5.07 ^####^	25.16 ± 3.02 ^####^
Total no. of cells (million/mouse)	-	75.92 ± 5.17	43.08 ± 3.56 ^###^	60.14 ± 3.61 ^NS^	17.68 ± 2.33 ^####^
Viable cells (%)	-	85.33 ± 3.89	68.15 ± 1.23 ^###^	73.5 ± 1.77 ^##^	65.27 ± 0.96 ^####^
Dead cells (%)	-	14.67 ± 2.1	31.85 ± 4.48 ^#^	26.5 ± 3.61 ^#^	35.74 ± 2.66 ^##^
Mean survival time (MST)	59.5	15	33	38	43.4
Increase in life span (ILs)	296.67	0	120	153.3	190

Values are represented as mean ± SE, *n* = 10 for each group. Means with different superscript asterisks in the same row differ significantly at *p* < 0.05. ^#^, *p* < 0.05; ^##^, *p* < 0.01; ^###^, *p* < 0.001; and ^####^, *p* < 0.0001 in comparison of treatment groups with the EAC group (Gp 2). NS, nonsignificant; EAC, Ehrlich ascites carcinoma; CIS, cisplatin; and EQ, ethoxyquin.

**Table 4:** Biochemical parameters in liver tissue and ascetic fluid of different groups of experimental mice.

Parameters	Gp 1 (Naive)	Gp 2 (EAC)	Gp 3 (EAC + CIS)	Gp 4 (EAC + EQ)	Gp 5 (EAC + CIS + EQ)
Hepatic catalase (mmole/min/mg tissue)	85.74 ± 1.91	35.27 ± 0.62 ****	47.05 ± 1.01 ^####^	77.22 ± 1.19 ^####^	59.91 ± 1.07 ^####^
Hepatic GST (µmole/min/mg tissue)	2.33 ± 0.07	1.09 ± 0.02 ****	1.38 ± 0.03 ^##^	2.16 ± 0.05 ^####^	2.23 ± 0.04 ^####^
Hepatic MDA (nmole/g tissue)	3.37 ± 0.29	23.58 ± 0.76 ****	17.35 ± 0.39 ^####^	9.76 ± 0.56 ^####^	13.43 ± 0.25 ^####^
Hepatic total protein (U/g tissue)	4.35 ± 0.09	2.12 ± 0.04 ****	2.58 ± 0.07 ^####^	3.1 ± 0.06 ^####^	2.85 ± 0.11 ^####^
Hepatic TAC (µmole/g tissue)	6.51 ± 0.22	3.47 ± 0.17 ****	4.75 ± 0.16 ^####^	5.39 ± 0.12 ^####^	6.23 ± 0.05 ^####^
EAC catalase (mmole/min/mg protein)	-	14.8 ± 0.25	22.28 ± 0.32 ^##^	31.26 ± 2.55 ^####^	26.0 ± 0.34 ^####^
EAC GST (μmole/min/mg protein)	-	1.13 ± 0.02	1.62 ± 0.06 ^####^	2.25 ± 0.03 ^####^	1.84 ± 0.05 ^####^
EAC MDA (nmole/mL)	-	46.35 ± 1.27	33.96 ± 0.45 ^####^	25.09 ± 0.34 ^####^	28.77 ± 0.17 ^####^
EAC total protein (µg/mL)	-	1.86 ± 0.1	2.2 ± 0.09 ^#^	3.92 ± 0.05 ^####^	3.01 ± 0.05 ^####^
EAC TAC (µmole/mL)	-	0.19 ± 0.02	0.71 ± 0.02 ^####^	0.89 ± 0.02 ^####^	0.82 ± 0.02 ^####^

Values are represented as mean ± SE, *n* = 10 for each group. Means with different superscript asterisks in the same row differ significantly at (*p* < 0.05). ****, *p* < 0.0001 in comparison of the EAC group (Gp 2) with the normal control group (Gp 1), and ^#^, *p* < 0.05; ^##^, *p* < 0.01; ^####^, *p* < 0.0001 in comparison of treatment groups with the EAC group (Gp 2). EAC, Ehrlich ascites carcinoma; CIS, cisplatin; EQ, ethoxyquin; GST, glutathione-s-transferase; TAC, total antioxidant capacity; and MDA, malondialdehyde.

## Data Availability

The data supporting the findings of this study are available upon request.
